# The Proposed Hospital Enquiry

**Published:** 1920-11-20

**Authors:** 


### THE PROPOSED HOSPITAL ENQUIRY.

After regarding the very different pictures of hospital solvency as drawn by Lord Knutsford, Mr. E. W. Morris, Mr. Verity, and Mr. Frank Mildmay, the public must find itself in a condition of complete bewilderment as to the true position. The first authority says that the system is dead, the second that it is merely sick, the third that it was never more alive, and the fourth that no proper diagnosis has yet been made.

Sir Arthur Stanley, in his letter to *The Times* on Tuesday, is inclined to agree with the last speaker, and suggests that what is most needed is a clear and authoritative statement as to the actual position of the hospitals, their needs, the extent to which they fulfil their mission, and the methods which must be adopted to secure the provision of adequate beds for the sick of the community in the Metropolis. He advocates the appointment of a small Committee by the Minister of Health to investigate the whole position and report, through the Minister, to Parliament. Then, he considers, we shall know where we are and what are the real problems with which we are to deal.

It seems that Sir Arthur is of opinion that this inquiry should be limited to London, and possibly he relies for information as to the provinces on the survey recently made by Sir Napier Burnett for the Red Cross and St. John's Societies. Sir Arthur Stanley has found strong evidence to show that the class which mostly benefits by the work of hospitals is responding to the call for further financial help. We believe this is quite true of the provinces, where for some reason the organisation of workpeople's contributions is much more skilfully carried out than in London. But in the Metropolis the system of patients' payments is proceeding splendidly, if we may judge from individual accounts, although no figures have been collected to show the aggregate yield.

It was but a week ago that we suggested that Dr. Addison was badly informed as to hospital economics. An excellent suggestion has now been made to him by the Treasurer of St. Thomas's Hospital, and we sincerely hope that he will act upon it. As Sir Arthur Stanley says, the real danger to the voluntary system is from within. Generalisation from one particular instance is always dangerous, but never more so than in the case of hospitals, where conditions and methods vary so much. As this journal has urged for many weeks, it is necessary to let the public know all. Sir Arthur agrees with us that if they have the data on which they can do a little clear thinking there is no doubt as to what will be the result.

The constitution of the proposed Committee of Enquiry is a matter requiring some attention. It is especially essential that some executive officers of hospitals should be included, and no members should be chosen from institutions which consider themselves to be on the verge of collapse, or which are unnaturally prosperous. Danger of calm judgment being affected by immediate surroundings should be avoided. We suggest that two executive officer-members of the Committee should be nominated by the Incorporated Association of Hospital Officers, and two members of the chairman or honorary worker class should be chosen by the British Hospitals Association. We are confident that the findings of a carefully chosen and authoritative Committee of Enquiry would be examined with the greatest care by Parliament and the public at large.

By means of a return of the financial position up to September 30, King Edward's Hospital Fund for London is in possession of quite recent information, and should be in a position materially to help the Committee of Enquiry.

